# Combination of Antistaphylococcal β-Lactam With Standard Therapy Compared to Standard Therapy Alone for the Treatment of Methicillin-Resistant *Staphylococcus aureus* Bacteremia: A Post Hoc Analysis of the CAMERA2 Trial Using a Desirability of Outcome Ranking Approach

**DOI:** 10.1093/ofid/ofae181

**Published:** 2024-04-25

**Authors:** Neta Petersiel, Joshua S Davis, Niamh Meagher, David J Price, Steven Y C Tong, Combination Antibiotics for MEthicillin Resistant Staphylococcus aureus (CAMERA2) Study Group, David C Lye, Dafna Yahav, Archana Sud, J Owen Robinson, Jane Nelson, Sophia Archuleta, Matthew A Roberts, Alan Cass, David L Paterson, Hong Foo, Mical Paul, Stephen D Guy, Adrian R Tramontana, Genevieve B Walls, Stephen McBride, Narin Bak, Niladri Ghosh, Benjamin A Rogers, Anna P Ralph, Jane Davies, Patricia E Ferguson, Ravindra Dotel, Genevieve L McKew, Timothy J Gray, Natasha E Holmes, Simon Smith, Morgyn S Warner, Shirin Kalimuddin, Barnaby E Young, Naomi Runnegar, David N Andresen, Nicholas A Anagnostou, Sandra A Johnson, Mark D Chatfield, Allen C Cheng, Vance G Fowler, Benjamin P Howden, Niamh Meagher, David J Price, Sebastiaan J van Hal, Matthew V N O Sullivan

**Affiliations:** Victorian Infectious Diseases Service, Royal Melbourne Hospital at the Peter Doherty Institute for Infection and Immunity, Melbourne, Victoria, Australia; Department of Infectious Diseases, University of Melbourne at the Peter Doherty Institute for Infection and Immunity, Melbourne, Victoria, Australia; Devision of Global and Tropical Health, Menzies School of Health Research and Charles Darwin University, Darwin, Northern Territory, Australia; Department of Infectious Diseases, John Hunter Hospital, Newcastle, New South Wales, Australia; Department of Infectious Diseases, University of Melbourne at the Peter Doherty Institute for Infection and Immunity, Melbourne, Victoria, Australia; Department of Infectious Diseases, University of Melbourne at the Peter Doherty Institute for Infection and Immunity, Melbourne, Victoria, Australia; Centre for Epidemiology and Biostatistics, Melbourne School of Population and Global Health, University of Melbourne, Melbourne, Victoria, Australia; Victorian Infectious Diseases Service, Royal Melbourne Hospital at the Peter Doherty Institute for Infection and Immunity, Melbourne, Victoria, Australia; Department of Infectious Diseases, University of Melbourne at the Peter Doherty Institute for Infection and Immunity, Melbourne, Victoria, Australia

**Keywords:** bacteremia, bloodstream infection, DOOR, methicillin-resistant *Staphylococcus aureus*, MRSA

## Abstract

**Background:**

Desirability of outcome ranking (DOOR) is an emerging approach to clinical trial outcome measurement using an ordinal scale to incorporate efficacy and safety endpoints.

**Methods:**

We applied a previously validated DOOR endpoint to a cohort of CAMERA2 trial participants with methicillin-resistant *Staphylococcus aureus* bacteremia (MRSAB). Participants were randomly assigned to standard therapy, or to standard therapy plus an antistaphylococcal β-lactam (combination therapy). Each participant was assigned a DOOR category, within which they were further ranked according to their hospital length of stay (LOS) and duration of intravenous antibiotic treatment. We calculated the probability and the generalized odds ratio of participants receiving combination therapy having worse outcomes than those receiving standard therapy.

**Results:**

Participants assigned combination therapy had a 54.5% (95% confidence interval [CI], 48.9%–60.1%; *P* = .11) probability and a 1.2-fold odds (95% CI, .95–1.50; *P* = .12) of having a worse outcome than participants on standard therapy. When further ranked according to LOS and duration of antibiotic treatment, participants in the combination group had a 55.6% (95% CI, 49.5%–61.7%) and 55.3% (95% CI, 49.2%–61.4%) probability of having a worse outcome than participants in the standard treatment group, respectively.

**Conclusions:**

When considering both efficacy and safety, treatment of MRSAB with a combination of standard therapy and a β-lactam likely results in a worse clinical outcome than standard therapy. However, a small benefit of combination therapy cannot be excluded. Most likely the toxicity of combination therapy outweighed any benefit from faster clearance of bacteremia.

## Teaser

In an analysis of the CAMERA2 trial using a desirability of outcome ranking approach, treatment of *Staphylococcus aureus* bloodstream infections with a combination of standard therapy with an antistaphylococcal penicillin likely results in a worse outcome than standard therapy alone.

## Graphical Abstract

**Graphical abstract:**
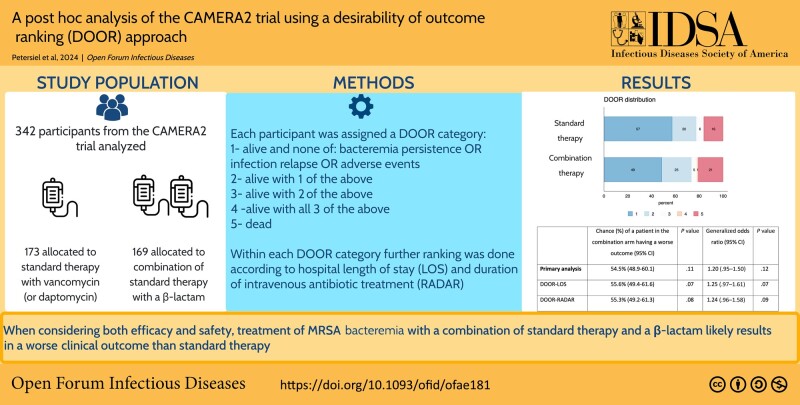
https://tidbitapp.io/tidbits/combination-of-anti-staphylococcal-lactam-with-standard-therapy-compared-to-standard-therapy-alonefor-the-treatment-of-mrsa-bacteraemia-a-post-hoc-analysis-of-the-camera2-trial-using-a-desirability-of-o-d43ca663-6e3b-4e79-8fe0-37def12033f5?utm_campaign=tidbitlinkshare&utm_source=IO

Methicillin-resistant *Staphylococcus aureus* bacteremia (MRSAB) is a life-threatening infection with limited treatment options. Given the high mortality and complication rate, several randomized clinical trials have investigated combination therapy as a strategy to improve patient outcomes [1–5]. CAMERA1 [1] and CAMERA2 [2] (Combination Antibiotics for MEthicillin Resistant *Staphylococcus aureus*) were 2 randomized clinical trials that compared the combination of standard therapy with β-lactams (flucloxacillin, oxacillin, or cefazolin) to standard therapy alone for the treatment of MRSAB. Both trials found a reduction in the duration of bacteremia in favor of the combination therapy, but no survival benefit. For CAMERA2 the composite primary outcome of 90-day mortality, persistent or relapsing bacteremia, and treatment failure was not statistically different between the 2 study arms. Notably, the study was stopped early due to high rates of acute kidney injury (AKI) in the combination group, an adverse event (AE) that was not captured by the primary outcome, but only as a secondary safety outcome.

Clinical trials often use a dichotomous primary outcome, assigning either “success” or “failure” for a given intervention over another. The US Food and Drug Administration had published guidance for industry development of new drugs for infections such as acute bacterial skin and skin structure infections [6] and hospital- and community-acquired pneumonia [7, 8], which suggests using the rate of treatment success or mortality as primary endpoints. While convenient to consider from a practitioner's point of view, such outcomes do not capture the broader patient experience. An intervention, while beneficial in some respects, might be harmful in others (ie, have detrimental side effects).

Composite outcomes, such as that used in CAMERA2 of 90-day mortality, persistent or relapsing bacteremia, and treatment failure, incorporate more outcomes and may increase the number of trial events and thus improve study power. However, each element of the composite outcome is weighted equally, when in reality this is not intuitively the case. For example, mortality should be considered worse than treatment failure with subsequent survival.

The desirability of outcome ranking (DOOR) approach was developed to address some of these issues [9–11]. The ranking uses an ordinal scale, incorporating both efficacy and safety endpoints, to consider the patient's outcome as a spectrum (good outcome without harm, to bad outcome and harm), rather than a binary endpoint. Each patient is designated a score according to a predetermined classification of clinical outcomes, including both efficacy and safety (ie, AEs). The outcomes are ranked in priority (ie, death is the worst, and more AEs are worse than fewer AEs). The distribution of DOOR is compared between the 2 study groups, and the chance of a patient in the intervention group having a better (or worse) outcome than a patient in the control group is calculated. For clinical trials aiming to optimize the use of antibiotics, response adjusted to the duration of antibiotic risk (RADAR) is a version of the DOOR methodology that incorporates the duration of antibiotic treatment into the patient outcome measure, assuming that a shorter duration is better. Using RADAR, every patient is given a DOOR category and then ranked again within each DOOR category, according to their duration of therapy.

Here, we analyze the CAMERA2 trial data within the DOOR framework, to determine whether there were differences between the standard therapy and the combination therapy groups with this broader consideration of health outcomes.

## METHODS

### Study Design

Methods for CAMERA2 have been previously published [2, 12]. In brief, CAMERA2 was an open label, multicenter, randomized clinical trial that compared treatment of MRSAB with standard therapy alone (either vancomycin or daptomycin), to treatment with standard therapy plus an antistaphylococcal β-lactam (antistaphylococcal penicillin or first-generation cephalosporin). The study enrolled participants in 4 countries (Australia, New Zealand, Singapore, and Israel) between 2015 and 2018. The primary endpoint was a composite of 90-day mortality, persistent bacteremia for 5 days, relapsing bacteremia >72 hours after culture sterilization, and a new metastatic focus of infection defined as a new sterile site culture >14 days from randomization. Secondary endpoints included all-cause mortality at days 14, 42, and 90, persistent bacteremia at day 2 and 5, AKI, microbiological relapse and failure, and duration of antibiotic treatment.

### Study Outcome and Definitions

We used a DOOR endpoint [10] that was developed by infectious diseases physicians and previously validated on 2 cohorts of MRSAB from the CAMERA1 trial, comparing monotherapy with vancomycin to the combination of vancomycin and β-lactam, and a second randomized controlled trial comparing vancomycin to trimethoprim-sulfamethoxazole. We applied a tailored DOOR endpoint, based on available data from the trial, to the CAMERA2 cohort of patients with MRSAB (Table 1).

**Table 1.** Components of the Original Desirability of Outcome Ranking (DOOR) Endpoint and the CAMERA2 DOOR Endpoint

Component	Suggested Original DOOR Endpoint	CAMERA2 DOOR
Treatment failure	Lack of global resolution of infection at 8 weeks	Primary treatment failure defined as persistent bacteremia on day 5
Infectious complications	Development of drug resistance OR Newly identified metastatic focus of infection OR Persistent or resistant *Staphylococcus aureus* BSI	Secondary treatment failure defined as either microbiological relapse (positive blood culture at least 72 hours after blood cultures sterilization) OR microbiological treatment failure (new metastatic infection diagnosed after day 14)
Ongoing symptoms	Ongoing symptoms that limit daily activities, with or without evidence of ongoing infection	None available
Adverse events	Grade 4 adverse events	Any degree of AKI, regardless of the outcome or clinical relevance for the patient, OR any adverse reaction (adverse events thought to be related to study drugs as reported by trial investigators)
Death	At end of follow-up (8 weeks)	Until 90 days

Abbreviations: AKI, acute kidney injury; BSI, bloodstream infection; CAMERA2, Combination Antibiotics for MEthicillin Resistant *Staphylococcus aureus*; DOOR, desirability of outcome ranking.

Each participant was assigned a score between 1 and 5 according to their clinical outcome:

1. Alive at day 90 without any of the following 3 features:
*(i)* Primary treatment failure: defined as persistent bacteremia (positive blood culture) at day 5; (*ii*) infectious complication: defined as either microbiological relapse (positive blood culture at least 72 hours after blood cultures sterilization) OR microbiological treatment failure (new metastatic infection diagnosed after day 14); (*iii*) AEs: defined as any degree of AKI, regardless of the outcome or clinical relevance for the patient, OR any adverse reaction (AEs thought to be related to study drugs as reported by trial investigators).
2. Alive with 1 of the above.
3. Alive with 2 of the above.
4. Alive with all 3 of the above.
5. Died.

AKI was defined according to a modified RIFLE (risk, injury, failure, loss, end-stage renal failure) criteria [13] as a 1.5-fold increase in creatinine at any time within the first 7 days, or a new need for renal replacement therapy between day 1 and day 90. Participants already undergoing hemodialysis or peritoneal dialysis at randomization were not eligible for the AKI endpoint.

### Statistical Analyses

All analyses were undertaken according to the intention-to-treat principle. No imputation was undertaken for missing values. Where one of the variables relating to DOOR components (90-day mortality, persistent or relapsing bacteremia, new metastatic infection, and creatinine) was missing, the participants were excluded from the primary analysis. A *P* value of <.05 was considered to indicate a statistically significant result.

#### Primary Analysis

As a primary analysis, participants assigned standard therapy were compared to participants assigned combination therapy. The chance that a participant treated with combination therapy had a worse DOOR ranking than a participant treated with standard therapy was assessed using a Mann-Whitney *U* test [10, 14]. Although the Mann-Whitney *U* test is mostly recognized as a nonparametric test to compare medians of continuous outcomes, it can be used to assess the probability that a randomly selected member from group A will have a better or worse outcome than a randomly selected member of group B [15]. We also calculated a generalized odds ratio (genOR), corresponding to the odds that a participant randomly selected from the combination therapy group will have a worse outcome than a participant randomly selected from the standard therapy group [16]. Both the Mann-Whitney and the genOR are considered conservative as pairs of participants with tied DOOR scores are maintained in the analysis and split evenly between having a better and worse outcome.

#### Secondary Analyses

We conducted 2 secondary analyses, where within each DOOR category patients were further ranked with a “tiebreaker.” In the first, referred to as DOOR-LOS, participants within each DOOR category (1­–4, excluding 5) were ranked based on their hospital length of stay (LOS). All participants within category 5 (ie, died) received the same lowest rank. LOS included acute care only, excluding days spent receiving antibiotics outside the inpatient settings such as hospital in the home (HITH) or outpatient parenteral antibiotic therapy (OPAT) services. For the second analysis, referred to as DOOR-RADAR, participants within DOOR categories 1–4 were ranked according to their total days of intravenous (IV) antibiotic treatment (including days spent in HITH and OPAT setting) using a DOOR/RADAR method [14]. The duration of intravenous vancomycin (or daptomycin) in the CAMERA2 trial [12] was at the clinicians’ discretion and according to Australian Therapeutic Guidelines and Infectious Diseases Society of America guidelines [17]. Again, participants in category 5 received the same lowest rank. We then compared participants by their treatment group using a Mann-Whitney *U* test.

#### Sensitivity Analyses

To explore the sensitivity of our primary analysis to our assumptions, we used 2 stricter AE definitions that are more clinically significant. First, we used a 2-fold increase in creatinine as a threshold to define AKI, which defines injury by RIFLE criteria [13] (compared to a 1.5-fold increase that indicates risk of AKI). For the second analysis, we included only AEs as recorded by trial investigators (who were not blinded to treatment assignment as this was an open-label trial).

We also performed sensitivity analyses to ensure the exclusion of participants with missing data did not introduce a bias. In the first, all participants missing a DOOR component were assigned the worst possible ranking of surviving patients. In the second, we explored the most extreme scenarios, where participants with missing data from the combination group received the worst rank whereas participants from the standard group received the best rank, and vice versa.

#### Subgroup Analysis

A subgroup analysis excluded participants on chronic dialysis at baseline, as patients on chronic dialysis are less likely to be represented in the “adverse event” category, which includes mostly AKI (the most commonly reported AE).

Analyses were performed using Stata version 17 (StataCorp LP) and R 4.2.2 software, using the genodds package to obtain genORs.

## RESULTS

The CAMERA2 trial enrolled 352 participants from Australia, New Zealand, Singapore, and Israel. The median age was 64 (interquartile range [IQR], 49–77) years, 241 (68%) were male, and 225 (64%) had healthcare-associated infections. Further patient characteristics can be found in the original report [2]. Data on 90-day mortality and persistent bacteremia were missing for 6 (1.7%, 3 from each arm) and 4 (1.1%, 2 from each arm) participants, respectively, leaving 342 participants for analysis, of which 173 (50.6%) were randomized to standard therapy and 169 (49.4%) were randomized to combination therapy.

### Primary Analysis

**Figure 1.**
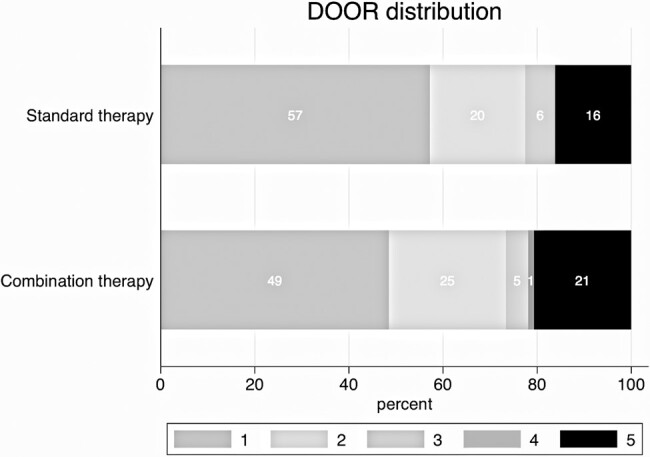
Desirability of outcome ranking (DOOR) distribution according to treatment groups: primary analysis. The DOOR is ranked from 1 (best) to 5 (worst). Percentages for each category are indicated within the bars.

The distribution of participants according to the 5 DOOR categories by treatment group is shown in Table 2 and Figure 1. The distribution of each DOOR component by treatment arm is presented in Supplementary Table 1. Participants in the combination therapy arm had a 54.5% (95% confidence interval [CI], 48.9%–60.1%; *P* = .11) probability of having a worse outcome than those in the standard therapy arm. The odds of being in a higher (worse) DOOR category was 1.2-fold (95% CI, .95–1.50; *P* = .12) greater for participants in the combination therapy arm compared to participants in the standard therapy arm.

**Table 2.** Distribution of Participants Within Desirability of Outcome Ranking Categories for the CAMERA2 Trial by Treatment Group^a^

DOOR Category	Standard Therapy, No. (%) (n = 173)	Combination Therapy, No. (%) (n = 169)
1	99 (57.2)	82 (48.5)
2	35 (20.2)	42 (24.9)
3	11 (6.4)	8 (4.7)
4	0	2 (1.2)
5	28 (16.2)	35 (20.7)

Abbreviations: CAMERA2, Combination Antibiotics for MEthicillin Resistant *Staphylococcus aureus*; DOOR, desirability of outcome ranking.

^a^Data on 2 of the DOOR components were missing for 10 participants, who were excluded from the primary analysis: Data on 90-day mortality were missing for 6 participants (1.7%, 3 from each arm), and data on persistent bacteremia were missing for 4 participants (1.1%, 2 from each arm).

### Secondary Analyses

#### DOOR-LOS

Participants were ranked according to their DOOR category and then their LOS. Information about LOS was available for 144 of 145 (99.3%) surviving participants in the standard therapy group and 132 of 134 (98.5%) of surviving participants in the combination therapy group. The median LOS for surviving participants was 22 (IQR, 13–38) days and 23 (IQR, 16–41) days in the standard and combination groups, respectively. Participants in the combination treatment group had a 55.6% (95% CI, 49.5%–61.7%) probability of having a worse outcome and a 1.25-fold odds (95% CI, .97–1.61) of being in a worse DOOR category compared to participants in the standard treatment group.

#### DOOR-RADAR

Finally, we conducted a DOOR-RADAR analysis, where participants were ranked according to overall days of IV antibiotic therapy within each DOOR category (1–5). Data on total days of IV antibiotics treatment were available for all participants. The median IV treatment duration for surviving participants was 28 (IQR, 16–41) days and 28 (IQR, 16–43) days in the standard and combination groups, respectively. Participants assigned to combination therapy had a 55.3% (95% CI, 49.2%–61.4%) probability and a 1.24-fold odds (95% CI, .96–1.58) of having a worse outcome than participants in the standard therapy arm.

### Sensitivity Analyses

#### Modified Definitions for AE

In the 2 sensitivity analyses with modified criteria for AKI, the DOOR outcome was similarly worse for participants receiving combination therapy as the primary analysis (Table 3, Supplementary Tables 2 and 3, and Supplementary Figures 1 and 2).

**Table 3.** Analyses of the CAMERA2 Trial Using a Desirability of Outcome Ranking Approach

Analysis	Chance of a Patient in the Combination Arm Having a Worse Outcome, % (95% CI)	*P* Value^a^	Generalized Odds Ratio (95% CI)	*P* Value^a^
Primary analysis	54.5 (48.9–60.1)	.11	1.20 (.95–1.50)	.12
Secondary analyses				
DOOR-LOS^b^	55.6 (49.4–61.6)		1.25 (.97–1.61)	
DOOR-RADAR	55.3 (49.2–61.3)		1.24 (.96–1.58)	
Sensitivity analyses				
Excluding dialysis patients	55.6 (49.4–61.6)		1.25 (.98–1.61)	
AKI defined as 2-fold increase in creatinine	53 (47.5–58.5)		1.13 (.90–1.41)	
Only AE reported by the trial's investigators	52.3 (46.8–57.8)		1.10 (.88–1.37)	
Missing data analyses				
Assuming the worst alive category	54.5 (48.9–60)		1.20 (.95–1.49)	
Assuming worst outcome for combination therapy and best outcome for standard therapy	55.4 (49.8–60.9)		1.24 (.99–1.56)	
Assuming best outcome for combination therapy and worst outcome for standard therapy	53.6 (48–59.1)		1.15 (.92–1.44)	

Abbreviations: AE, adverse event; AKI, acute kidney injury; CAMERA2, Combination Antibiotics for MEthicillin Resistant *Staphylococcus aureus*; CI, confidence interval; DOOR, desirability of outcome ranking; LOS, length of stay; RADAR, response adjusted for duration of antibiotic risk.

^a^ *P* values were not calculated for secondary, sensitivity, and missing data analyses as these are exploratory analyses.

^b^Analysis of the DOOR-LOS endpoint included 144 and 132 surviving participants from the standard therapy and combination therapy arms, respectively, for whom LOS data were available. One participant from the standard therapy arm and 2 participants from the combination therapy arm had missing LOS data and were excluded from this analysis.

#### Missing Data Analyses

Analyses exploring the most extreme scenarios—including all participants with missing data as either receiving best or worst outcomes in each group—yielded similar results to the primary analysis (Table 3).

#### Subgroup Analyses

The subgroup of participants not receiving chronic hemodialysis treatment consisted of 143 participants in the standard group and 145 participants in the combination group. There was a 55.6% (95% CI, 49.4%–61.7%) probability of a participant in the combination group having a worse outcome than in the standard group (Supplementary Table 4 and Supplementary Figure 3). Similarly, the odds of having a worse outcome were 1.25-fold (95% CI, .97–1.61) greater for participants in the combination therapy group compared to standard therapy.

## DISCUSSION

In this post hoc analysis using a DOOR approach, treatment of MRSAB with a combination of standard therapy and a β-lactam was associated with point estimates of 54.5% (95% CI, 48.9%–60.1%; *P* = .11) chance and a 1.2-fold odds (95% CI, .95–1.50; *P* = .12) of a worse clinical outcome than treatment with standard therapy alone. Although not reaching statistical significance (*P* > .05) and thus not being able to entirely exclude a benefit of combination therapy, this finding of poorer outcomes was consistent using the classic DOOR outcome and when taking into account the length of hospital stay and duration of treatment. The findings were robust to analytical assumptions, as demonstrated in the sensitivity analyses.

The results of the DOOR analyses provide a different emphasis and viewpoint compared to the originally reported results using the primary composite outcome measure [2]. Whereas the primary composite outcome in CAMERA2 was suggestive of a better outcome in the combination group (35% in the combination group and 39% in the standard group met the primary composite outcome, *P* = .42) the analysis according to the DOOR suggests an increased probability of worse outcomes in the combination group. This difference was driven by the higher rate of AKI and AE in the combination group. Even though the CAMERA2 trial was stopped early due to the increased risk of AKI in participants who received combination therapy, the primary endpoint did not reflect these events, which were captured only as a safety outcome.

Compared to classic binary outcomes, the global patient outcome as assessed by the DOOR scale balances clinically significant efficacy endpoints with safety endpoints. Since it was first described by Evans et al in 2015 [14], several studies have used DOOR to measure outcomes [18–23], and a recent narrative review summarized its utility in infectious diseases clinical trials [11]. One of the challenges of creating a DOOR endpoint is deciding which components to use as part of the ranking. Ideally, the components of the DOOR would be clinically significant for the patient, reflecting treatment success as well as the quality of life. While in the case of life-threatening infections such as MRSAB, the best (alive and well) and worst (deceased) categories are usually easy to decide upon, the components of the intermediate categories are often less straightforward. Doernberg et al [10] developed a DOOR endpoint by administering a survey to 43 infectious diseases specialists and asking them to rank the desirability of different outcomes of patients with *S aureus* bloodstream infections. The outcomes chosen to generate the DOOR endpoint were treatment failure, infectious complications, ongoing symptoms, and grade 4 AEs. Interestingly, clinicians tended to agree about which scenarios had the best and worst outcomes, but distinguishing intermediate outcomes proved more difficult and there was no agreement as to which of the DOOR components were more important than the others. They then applied the ordinal DOOR endpoint to 2 previously published MRSAB randomized trials, CAMERA1 (n = 60), comparing standard therapy to combination therapy, and a second randomized trial by Paul et al comparing trimethoprim-sulfamethoxazole (TMP-SMX) to vancomycin (n = 91) [24]. For both trials, the DOOR outcome aligned with the original results of the studies, which were neutral for vancomycin compared to combination in CAMERA1, and in favor of vancomycin in the TMP-SMX trial.

Strengths of our study include large sample size, performing multiple analyses with various approaches to defining the DOOR endpoint, and the use of sensitivity analyses to explore how choices made in the analyses may impact findings. We used a similar DOOR outcome to the one developed and validated by Doernberg et al [10], applying it to the largest cohort of MRSAB patients to date. As stated above, the DOOR outcome in our analyses did not echo the original trial's outcome, but arguably provided a more accurate reflection of the global outcome of participants in the trial given the incorporation of safety endpoints.

This was a post hoc analysis, and as such has limitations. First, we could only use data that were gathered as part of the original trial and therefore our DOOR endpoint did not include quality-of-life measures that were not collected but are important to patients. Therefore, we could not explore a broader range of outcomes. The recently launched *Staphylococcus aureus* Network Adaptive Platform (SNAP) randomized clinical trial [25], which is in the early stages of recruitment at the time of writing, assesses different treatment options for *S aureus* bacteremia and uses an ordinal outcome as a secondary outcome, incorporating data on functional capacity at 90 days. An ongoing study comparing dalbavancin to standard of care for complicated *S aureus* bacteremia (Dalbavancin as an Option for Treatment of *Staphylococcus aureus* bacteremia [DOTS] trial) [26] is the first to use DOOR as a primary outcome measure in a randomized clinical trial and includes the absence of bacteremia-related signs and symptoms at 70 days to define clinical success. Second, some of the DOOR components may not be clinically significant to the patient, such as a 1.5-fold elevation in creatinine level (defined as “risk” in the RIFLE criteria [13]). We tried to address this performing sensitivity analyses using stricter definitions for adverse effects, such as 2-fold increase in creatinine (defined as “injury”), and by incorporating the LOS, which could be influenced by the clinical impact of the particular outcome the participant experienced. Furthermore, our prevalidated endpoint was created by a group of infectious diseases specialists and reflects what they considered to be clinically significant outcomes. Future studies may add patients’ experiences and what they consider to be a “good” outcome to this ranking. Third, the DOOR endpoint applies equivalent weight to all the components (apart from death), similar to composite endpoints, when not all of these outcomes carry the same significance. In our DOOR endpoint, a participant with a single clinically significant event (eg, relapse of their infection) would be given the same ranking as a participant with a single minimally impactful event, such as stage 1 AKI. Another option would be instead to rank the different components by order of their clinical relevance, creating a hierarchy of outcomes. Such approach had been suggested by Pocock et al, who developed the “win ratio” [27], whereby each pair of participants from the intervention and control groups is compared in a stepwise process according to a predefined hierarchical outcome. Such an approach has recently been applied to the MERINO trial (Meropenem versus Piperacillin-tazobactam for definitive treatment of bloodstream infections due to ceftriaxone non-susceptible *Escherichia Coli* and *Klebsiella Spp*) [28]. While this approach emphasizes clinical priorities, it does not account for the possibility of multiple events happening to a single participant. Finally, the CAMERA2 trial was stopped early due to increased AKI in the combination therapy arm. The lower number of participants reduces the power of the study to detect a difference between arms for both the original analysis as well as this DOOR analysis.

In summary, in contrast to the findings for the composite endpoint from the original CAMERA2 trial, the DOOR endpoint indicates that combination therapy with vancomycin or daptomycin and a β-lactam likely results in poorer outcomes compared to standard therapy. Despite not reaching statistical significance (*P* < .05), the direction of the effect was consistent across all analyses and was robust to changes in DOOR definition. The likely poorer outcomes in the combination therapy group were driven mostly by higher rates of AEs, mainly AKI.

## Supplementary Data

Supplementary materials are available at *Open Forum Infectious Diseases* online. Consisting of data provided by the authors to benefit the reader, the posted materials are not copyedited and are the sole responsibility of the authors, so questions or comments should be addressed to the corresponding author.

## Supplementary Material

ofae181_Supplementary_Data

## Notes

***Acknowledgments.*** The CAMERA2 study group includes the following: David C. Lye, Dafna Yahav, Archana Sud, J. Owen Robinson, Jane Nelson, Sophia Archuleta, Matthew A. Roberts, Alan Cass, David L. Paterson, Hong Foo, Mical Paul, Stephen D. Guy, Adrian R. Tramontana, Genevieve B. Walls, Stephen McBride, Narin Bak, Niladri Ghosh, Benjamin A. Rogers, Anna P. Ralph, Jane Davies, Patricia E. Ferguson, Ravindra Dotel, Genevieve L. McKew, Timothy J. Gray, Natasha E. Holmes, Simon Smith, Morgyn S. Warner, Shirin Kalimuddin, Barnaby E. Young, Naomi Runnegar, David N. Andresen, Nicholas A. Anagnostou, Sandra A. Johnson, Mark D. Chatfield, Allen C. Cheng, Vance G. Fowler Jr, Benjamin P. Howden, Niamh Meagher, David J. Price, Sebastiaan J. van Hal, and Matthew V. N. O Sullivan.

***Patient consent.*** Because this study was a secondary analysis of previously collected data, reconsent of participants was not required. As part of the initial trial, participants provided written informed consent, as described in the primary manuscript by Tong et al. [2].

***Financial support.*** The original CAMERA2 trial was funded by competitive grants from the Australian National Health and Medical Research Council (grant number 1078930) and the Singapore National Medical Research Council (grant number 0001-001094) and by seed funding from the Ramiciotti Foundation (grant number ES2014/079).
