# Supplementary material for: Combination of Antistaphylococcal β-Lactam With Standard Therapy Compared to Standard Therapy Alone for the Treatment of Methicillin-Resistant Staphylococcus aureus Bacteremia: A Post Hoc Analysis of the CAMERA2 Trial Using a Desirability of Outcome Ranking Approach
Source: Open Forum Infect Dis. 2024 Apr 25;11(5):ofae181. doi: 10.1093/ofid/ofae181 (PMC11065345; doi:10.1093/ofid/ofae181)
Supplement: ofae181_Supplementary_Data [file ofae181_supplementary_data.docx]

**Supplementary appendix**

**Table S1**: Distribution of the DOOR components by treatment group

**Table S2**: The distribution of DOOR categories for the CAMERA2 trial by treatment group, using a stricter definition for adverse events.

**Table S3**: The distribution of DOOR categories for the CAMERA2 trial by treatment group, adverse events include all reported AE by trial’s investigators.

**Table S4**: The distribution of DOOR categories for the CAMERA2 trial by treatment group, excluding participants on chronic hemodialysis.

**Figure S1**: DOOR distribution according to treatment groups, using a stricter definition for adverse events. Adverse events were defined as a 2-fold increase in creatinine (RIFLE definition for acute kidney injury) or AE as reported by trial’s investigators

**Figure S2**: DOOR distribution according to treatment groups. AE included only those reported by the trial’s investigators.

**Figure S3**: DOOR distribution according to treatment groups excluding patients on chronic dialysis treatment.

**Table S1**: Distribution of the DOOR components by treatment group

| DOOR component | Standard therapy, n(%) N=173 | Combination therapy, n(%) N=169 |
| --- | --- | --- |
| Primary treatment failure (persistent bacteremia at trial day 5) | 34 (19.7) | 19 (11.2) |
| Infectious complication   - Microbiological relapse - Microbiological treatment failure | 25 (14.5)^1^  18 (10.4)  16 (9.2) | 21 (12.4)^1^  14 (8.3)  15 (8.9) |
| Adverse events   - Any degree of AKI - Adverse events reported by investigators | 15 (8.7)  8 (4.6)  7 (4) | 44 (26)^2^  34 (20.1)  23 (13.6) |
| 90-days mortality | 28 (16.2) | 35 (20.7) |

Abbreviations: DOOR, desirability of outcome ranking; AKI, acute kidney injury

^1^ 9 participants in the standard therapy arm and 8 participants in the combination therapy arm had both microbiological relapse and microbiological treatment failure.

^2^13 participants in the combination therapy arm had both AKI and AE reported by study investigators.

Sensitivity analysis

**Table S2**: The distribution of DOOR categories for the CAMERA2 trial by treatment group, using a stricter definition for adverse events.

Adverse events were defined as a 2-fold increase in creatinine (RIFLE definition for acute kidney injury) or AE as reported by trial’s investigators. Participants who received combination therapy with a β-lactam had a 53% (47.5-58.6); p=0.28) chance of having a worse outcome than patients on standard therapy.

| DOOR category | Standard therapy, n (%) | Combination therapy, n (%) |
| --- | --- | --- |
| 1 | 101 (58.3) | 90 (53.2) |
| 2 | 34 (19.7) | 35 (20.7) |
| 3 | 10 (5.8) | 7 (4.1) |
| 4 | 0 | 2 (1.2) |
| 5 | 28 (16.2) | 35 (20.7) |
| Total | 173 | 169 |

Abbreviations: DOOR, desirability of outcome ranking; CAMERA2, Combination Antibiotics for Methicillin Resistant *Staphylococcus aureus*; RIFLE, Risk Injury Failure Loss End-stage renal failure; AE, adverse events.

**Table S3**: The distribution of DOOR categories for the CAMERA2 trial by treatment group, adverse events include all reported AE by trial’s investigators. Participants who received combination therapy with a β-lactam had a 52.3% (46.8 - 57.8); p=0.40) chance of having a worse outcome than participants on standard therapy.

| DOOR category | Standard therapy, n (%) | Combination therapy, n (%) |
| --- | --- | --- |
| 1 | 103 (59.6) | 95 (56.2) |
| 2 | 32 (18.5) | 30 (17.8) |
| 3 | 10 (5.8) | 8 (4.7) |
| 4 | 0 | 1 (0.6) |
| 5 | 28 (16.2) | 35 (20.7) |
| Total | 173 | 169 |

Abbreviations: DOOR, desirability of outcome ranking; CAMERA2, Combination Antibiotics for Methicillin Resistant *Staphylococcus aureus*; AE, adverse events.

Subgroup analysis

**Table S4**: The distribution of DOOR categories for the CAMERA2 trial by treatment group, excluding participants on chronic haemodialysis.

| DOOR category | Standard therapy, n (%) | Combination therapy, n (%) |
| --- | --- | --- |
| 1 | 83 (58) | 65 (44.8) |
| 2 | 24 (16.8) | 40 (27.6) |
| 3 | 9 (6.3) | 7 (4.8) |
| 4 | 0 | 2 (1.4) |
| 5 | 27 (18.9) | 31 (21.4) |
| Total | 143 | 145 |

Abbreviations: DOOR, desirability of outcome ranking; CAMERA2, Combination Antibiotics for Methicillin Resistant *Staphylococcus aureus*.

**Figure S1**: DOOR distribution according to treatment groups, using a stricter definition for adverse events. Adverse events were defined as a 2-fold increase in creatinine (RIFLE definition for acute kidney injury) or AE as reported by trial’s investigators.


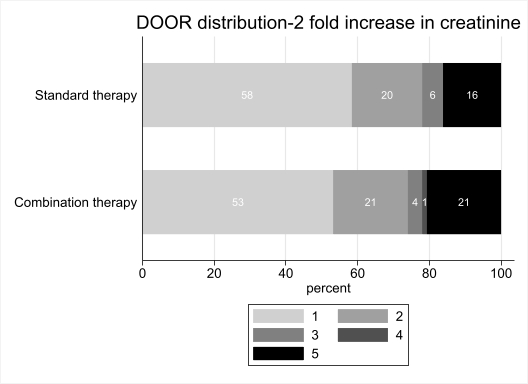


Abbreviations: DOOR, desirability of outcome ranking; RIFLE, Risk Injury Failure Loss End-stage renal failure; AE, adverse events.

**Figure S2**: DOOR distribution according to treatment groups. AE included only those reported by the trial’s investigators.


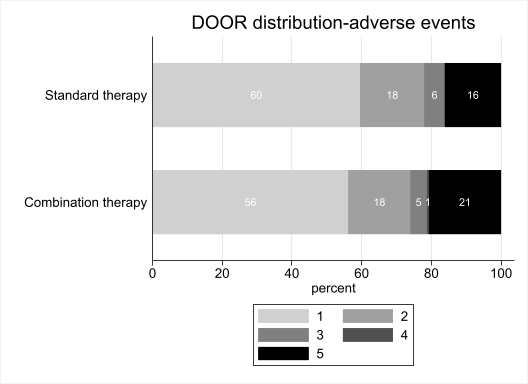


Abbreviations: DOOR, desirability of outcome ranking; AE adverse events.

**Figure S3**: DOOR distribution according to treatment groups excluding patients on chronic dialysis treatment.


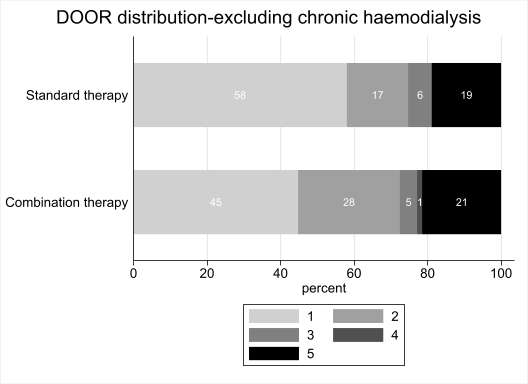


Abbreviations: DOOR, desirability of outcome ranking.
